# Advantages of BioMatrix respiratory gating in free-breathing three-dimensional magnetic resonance cholangiopancreatography: a prospective comparative study

**DOI:** 10.1186/s13244-025-02023-4

**Published:** 2025-06-27

**Authors:** Qing Yang, Xueyi Ding, Qiuyang Guo, Yifan Tang, Jianyu Lin, Yantu Huang, Mengxiao Liu, Junqiang Lei

**Affiliations:** 1 Department of Radiology, The First Hospital of Lanzhou University, Lanzhou, China; 2 Department of Medical Imaging, Anhui Medical University Anqing Medical Center (Anqing Municipal Hospital), Anqing, China; 3 Department of Magnetic Resonance, Siemens Shenzhen Magnetic Resonance Ltd., Shenzhen, China; 4 MR Research Collaboration Team, Diagnostic Imaging, Siemens Healthineers Ltd., Shanghai, China; 5 Gansu Provincial Clinical Medical Center for Radiological Imaging, Lanzhou, China

**Keywords:** Magnetic resonance cholangiopancreatography, BioMatrix, Free breathing, Image quality

## Abstract

**Objectives:**

To compare the image acquisition time, total examination time, image quality, and technical reliability of three free-breathing MRCP techniques: BioMatrix-triggered (BM-MRCP), respiratory-gating triggered using respiratory bellows (RG-MRCP), and navigator-triggered (NT-MRCP).

**Methods:**

A prospective intra-individual comparison was performed in 47 patients undergoing 3.0-T MRCP for suspected pancreatic and biliary diseases. Two patients with technique adaptability limitations were included in the reliability analysis as “technical failures.” For primary analyses, data from 45 patients completing all three techniques were used. Image quality was evaluated by three blinded radiologists (experience: 5, 10, 16 years). Statistical analysis included Friedman tests with Bonferroni correction (*p* < 0.0167).

**Results:**

Median total examination times were significantly shorter for BM-MRCP (218 [48] seconds) compared to RG-MRCP (228 [56] seconds) and NT-MRCP (259 [53] seconds) (*p* < 0.05). BM-MRCP and RG-MRCP had comparable image acquisition times, both significantly faster than NT-MRCP (*p* < 0.05). BM-MRCP provided superior image quality for key anatomical structures (*p* < 0.05), higher SNR, and CNR compared to RG-MRCP and NT-MRCP (*p* < 0.05). Image contrast showed no significant differences (*p* > 0.05). Two patients experienced failures with RG-MRCP or NT-MRCP due to breathing issues, while BM-MRCP had no failures.

**Conclusion:**

BM-MRCP significantly reduces examination times while achieving superior image quality and technical reliability. Its integration into clinical workflows enhances efficiency, reduces technician workload, and improves patient-centered imaging.

**Critical relevance statement:**

BioMatrix-gating 3D-MRCP enhances imaging efficiency and diagnostic accuracy for the biliary and pancreatic duct systems. By reducing scan times and improving workflow, it supports patient comfort and compliance. Its simplicity and reliability also make it ideal for high-throughput clinical settings.

**Key Points:**

- BioMatrix-triggered (BM)-MRCP shortens examination time, aiding patients with compliance or limitations.
- BM-MRCP offers superior image quality with reduced motion artifacts and higher clarity.
- BM respiratory sensors streamline workflows, boost reliability, and enhance patient comfort.

**Graphical Abstract:**

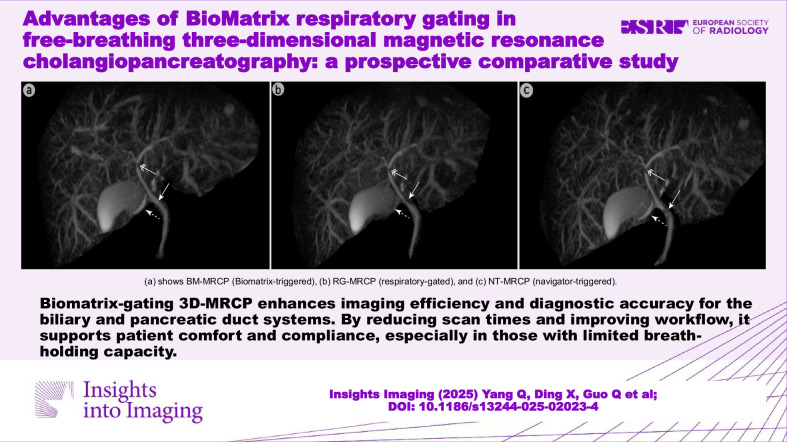

## Introduction

Magnetic resonance cholangiopancreatography (MRCP) is a non-invasive imaging technique that uses heavily T2-weighted sequences to generate high-contrast imaging of fluid-filled structures, such as the pancreatic and biliary ducts. It facilitates the evaluation of the morphology, patency, and pathological changes in the pancreatic and biliary duct systems [1, 2]. Given its safety and accuracy, MRCP has become a non-invasive alternative to endoscopic retrograde cholangiopancreatography (ERCP) [3–5].

Early two-dimensional MRCP was limited by spatial resolution. However, the development of three-dimensional scanning sequences has significantly improved image quality and lesion visualization [6–8]. These sequences enable isotropic imaging through multiplanar reconstruction, addressing previous limitations, although prolonged acquisition times remain susceptible to motion artifacts [9, 10]. Various breath-holding techniques, such as rapid spin-echo and compressed sensing, have reduced scan times to under 20 s [11–14]. However, their use is limited to patients with impaired pulmonary function or frailty, who may struggle to comply with breath-holding requirements.

Respiratory-gated techniques provide an alternative for patients unable to hold their breath [15]. Common free-breathing MRCP techniques include respiratory belt gating (RG) and diaphragm navigator triggering (NT). RG requires external device installation, while NT necessitates precise adjustment of the navigator position and a pre-learning process. Recent efforts have also explored compressed sensing MRCP combined with contact-free physiological monitoring (CFPM). For example, He et al [16] employed a camera-based optical motion detection system (VitalEye, Philips Healthcare) as the CFPM solution and demonstrated the feasibility of CS-CFPM-MRCP in patients with suspected pancreaticobiliary disorders. However, such vision-based systems may require a fixed visual field and can be affected by lighting or patient positioning.

In contrast, Siemens’ BioMatrix (BM) technology incorporates integrated respiratory sensors into the MR system, detecting respiratory signals in real time through impedance changes in the embedded RF coils [17]. This sequence-independent solution eliminates the need for external devices, calibration, or line-of-sight, offering potential advantages in workflow integration and robustness across various patient populations. Despite its technical potential, no prior studies have systematically investigated the impact of BioMatrix gating on 3D-MRCP scan time, image quality, or overall clinical workflow.

Therefore, this prospective study aims to compare three respiratory-gating techniques for free-breathing 3D-MRCP—BioMatrix-triggered (BM-MRCP), respiratory-gating triggered using respiratory bellows (RG-MRCP), and navigator-triggered (NT-MRCP)—with a focus on image quality, scan efficiency, artifact control, and technical reliability.

## Methods

This prospective study was approved by the ethics committee, and all patients provided written informed consent.

The primary objective of this study is to assess and compare the differences in image quality, signal-to-noise ratio (SNR), contrast-to-noise ratio (CNR), and scan time across the three MRCP techniques: BM-MRCP, RG-MRCP, and NT-MRCP. Secondary aims included evaluating BM-MRCP’s workflow benefits, artifact reduction, and visualization of key anatomical structures: the left/right peripheral hepatic ducts, left/right hepatic ducts, common hepatic duct, common bile duct, cystic duct, gallbladder, and the main pancreatic duct.

### Patients

This prospective study enrolled patients who underwent MRCP examination at a tertiary referral hospital between July and November 2024.

Inclusion criteria were: (1) a clinical indication for MRCP to evaluate pancreatic or biliary tract diseases; and (2) the ability to undergo at least one of the three MRCP techniques (BM-MRCP, RG-MRCP, or NT-MRCP) during a single session.

**Fig. 1:**
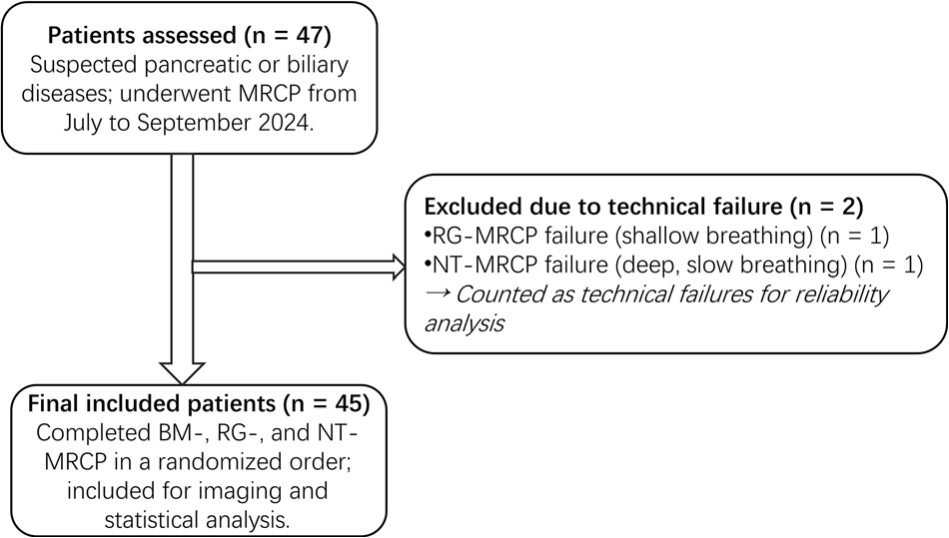
Patient enrollment process. MRCP, magnetic resonance cholangiopancreatography; BM-MRCP, BioMatrix MRCP; RG-MRCP, respiratory-gating triggered using respiratory bellows; NT-MRCP, navigator-triggered MRCP

Exclusion criteria were: (1) Pediatric patients (age < 18 years); (2) contraindications to MRI examination (e.g., metallic implants, severe claustrophobia); (3) inability to complete any technique due to noncompliance or technical issues; (4) history of pancreaticobiliary interventions that severely compromise biliary anatomy or preclude MRCP evaluation (e.g., extensive surgical reconstruction, metallic biliary stents) (Fig. 1).

### Equipment

All MRI scans were performed on a 3.0-T scanner (MAGNETOM Vida, Siemens Healthineers, Erlangen, Germany) equipped with an 18-channel body coil and a 32-channel spine array coil.

### Patient preparation

Patients were instructed to fast for at least 4 h before the examination and to avoid antispasmodic medications. Before each scan, the technician provided standardized breathing training. This training included practicing inspiration and expiration according to the scanning navigator rhythm and completing three stable breathing cycles. Training duration was recorded using a stopwatch.

### Scanning protocol

All patients underwent 3D-MRCP scans using three gating methods (BM, RG, NT) under free-breathing conditions in a randomized order. A T2-weighted 3D-SPACE sequence was used with the following uniform sequence parameters to ensure consistent image quality and acquisition times: Field of View (FOV): 240 × 320 mm²; Matrix: 384 × 384; Slice thickness: 1.4 mm; Spatial resolution: 0.4 × 0.4 × 1.2 mm³; Repetition time (TR): 1900 ms; Echo time (TE): 400 ms; Flip angle: 100°. Turbo Factor: 180; Acceleration mode: GRAPPA, APE-3.

### Time measurement and definitions

BM-MRCP total examination time was defined as the sum of breathing training time and BM-MRCP image acquisition time. RG-MRCP total examination time was the sum of respiratory belt installation time, breathing training time, and RG-MRCP image acquisition time. NT-MRCP total examination time was the sum of breathing training time, navigator setup time, navigator learning time, and NT-MRCP image acquisition time.

Image acquisition times were automatically recorded in seconds from the DICOM tag (0051,100A) by the scanner. The NT navigator learning time was calculated as “trigger period (seconds) × number of respiratory cycles,” with both values retrieved from the DICOM tag “Trig. period” and navigator images.

### Image analysis

All MRI images were independently evaluated by three radiologists specializing in abdominal imaging, with 5, 10, and 16 years of experience, respectively. To ensure consistent interpretation of the scoring criteria, all radiologists underwent standardized training before the image analysis. Both qualitative and quantitative analyses were performed for all subjects.

The images were assessed in a randomized order, and the radiologists were blinded to the specific imaging techniques and clinical information. Randomization was implemented to reduce potential bias related to the order of image evaluation. A washout period of 3 days was introduced between assessments to minimize memory bias, ensuring that each radiologist independently evaluated each imaging technique without being influenced by prior assessments.

### Qualitative scoring metrics

Scores were assigned using a four-point scale, with radiologists trained to ensure a consistent understanding of the scoring criteria. The final score was the average of the three radiologists’ scores. Detailed scoring standards are as follows:

1. Structural Evaluation: Clarity of the right and left peripheral hepatic ducts, right and left hepatic ducts, common hepatic duct, common bile duct (CBD), cystic duct, gallbladder, and main pancreatic duct; 1 = structure not visible, no detail, non-diagnostic; 2 = structure visible but details are blurred, limited diagnostic value; 3 = structure partially visible with artifacts; 4 = all structures are clearly visible, with distinct details.
2. Artifact Assessment: Degree of artifacts affecting image quality; 1 = severe artifacts, impairing diagnosis; 2 = moderate artifacts, still diagnosable; 3 = mild artifacts; 4 = no artifacts.
3. Overall Image Quality: Overall diagnostic usability of the images; 1 = poor; 2 = fair; 3 = good; 4 = excellent.

### Quantitative analysis

Signal-to-noise ratio (SNR), contrast, and contrast-to-noise ratio (CNR) were evaluated quantitatively. These metrics were calculated based on the mean signal intensity (SI) of the CBD, surrounding tissues, and liver, as well as the standard deviation (SD) of the background noise. The data were averaged across the three observers. The regions of interest (ROIs) were defined as follows: CBD, A homogeneous, artifact-free area in the mid-section of the CBD on a representative slice, with a circular ROI of at least 5 mm²; Surrounding Tissues, A uniform, artifact-free area, with an ROI of at least 20 mm²; Liver, A 20 mm² ROI in an area free of vessels and artifacts (Supplementary Fig. 1). SNR, contrast, and CNR were calculated using standard formulas (Eqs. 1–3), as described in references [11, 12].

1 $${SNR}=\frac{{{SI}}_{{CBD}}}{{{SD}}_{{CBD}}}$$

2 $${Contrast}=\frac{{{SI}}_{{CBD}}-{{SI}}_{{Periductal\; tissues}}}{{{SI}}_{{CBD}}+{{SI}}_{{Periductal\; tissues}}}$$

3 $${CNR}=\frac{{{SI}}_{{CBD}}-{{SI}}_{{Liver}}}{\sqrt{\left[{\left({{SD}}_{{CBD}}\right)}^{2}+{\left({{SD}}_{{Liver}}\right)}^{2}\right]/2}}$$

### Statistical analysis

The sample size for this study was estimated using G*Power (version 3.1.9.7, Heinrich Heine University, Düsseldorf, Germany). software. Based on an expected effect size of 0.5 for image quality scores, a significance level of 0.05, and a power of 80%, the estimated minimum sample size was 22 patients. Therefore, the final sample size in this study was 45 patients, which is adequate for statistical analysis.

Statistical analysis was performed using IBM SPSS Statistics for Windows, version 26.0 (IBM Corp.), and data visualization was conducted with Origin 2024 (OriginLab Corp.). All tests were two-sided, with *p* < 0.05 considered statistically significant.

Data following a normal distribution were presented as mean ± standard deviation (SD), while non-normally distributed data were described as median [interquartile range, Median (IQR)]. The normality of data was assessed using the Kolmogorov–Smirnov and Shapiro–Wilk tests.

The consistency of image quality scores assigned by the three radiologists was evaluated using the intraclass correlation coefficient (ICC). The criteria for consistency were as follows: Poor consistency: ICC < 0.40; Moderate consistency: 0.40 ≤ ICC < 0.75; Good consistency: 0.75 ≤ ICC < 0.90; Excellent consistency: ICC ≥ 0.90. The 95% confidence interval (CI) for the ICC was also calculated.

Comparisons among the three MRCP techniques (BM-MRCP, RG-MRCP, and NT-MRCP) were performed using the Friedman test. The analyzed variables included: Image acquisition time; Total examination time; Duct visibility scores; Artifact scores; Overall image quality scores; Signal-to-noise ratio (SNR); Contrast; Contrast-to-noise ratio (CNR). For variables with significant differences identified by the Friedman test, post hoc pairwise comparisons with Bonferroni correction (adjusted *p* < 0.0167) were conducted to determine specific intergroup differences.

## Results

### Patient characteristics

A total of 47 patients were enrolled in this study, comprising 29 males and 18 females, with a mean age of 41.64 ± 14.05 years (range: 22–72 years).

Among these, two patients could not complete all three MRCP techniques and were included as “technical failures”: one patient was unable to complete RG-MRCP due to shallow-slow breathing, and one failed NT-MRCP due to deep-slow breathing and prolonged scan time.

Thus, 45 patients successfully completed BM-MRCP, RG-MRCP, and NT-MRCP sequences and were included in the final comparative analysis. All MRCP scans were performed in a randomized order during a single examination session. The acquired images were categorized into three groups: BM-MRCP, RG-MRCP, and NT-MRCP.

Final diagnoses were established through clinical evaluation, surgical pathology, and advanced imaging (e.g., liver-specific contrast-enhanced T1-weighted cholangiopancreatography), combined with clinical records. The diagnostic spectrum included six normal cases, 30 cases of gallbladder or bile duct stones, five cases of gallbladder adenomyomatosis, one case post-cholecystectomy, two cases of intraductal papillary mucinous neoplasm (IPMN), and one case of congenital focal biliary stricture.

### Image acquisition time and total examination time

**Fig. 2:**
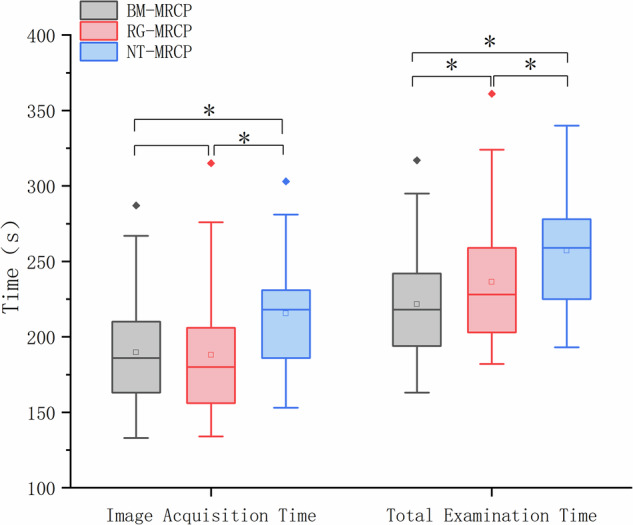
Comparisons of image acquisition and total examination times. BM-MRCP showed significantly shorter acquisition and examination times than NT-MRCP (*p* < 0.001), and shorter total examination time than RG-MRCP. One outlier was present in each group. MRCP, magnetic resonance cholangiopancreatography; BM-MRCP, BioMatrix MRCP; RG-MRCP, respiratory-gated MRCP; NT-MRCP, navigator-triggered MRCP

The results for image acquisition time and total examination time are summarized in Table 1 and Fig. 2. Under free-breathing conditions, the image acquisition times for BM-MRCP, RG-MRCP, and NT-MRCP were 186 (47) seconds, 180 (50) seconds, and 218 (45) seconds, respectively. There was no significant difference in image acquisition time between BM-MRCP and RG-MRCP, while NT-MRCP was significantly longer than the other two methods.

**Table 1:** Comparison of average MRCP acquisition time and total examination time

	BM-MRCP	RG-MRCP	NT-MRCP	*p*-value	*p*-value^a^	*p*-value^b^	*p*-value^c^
Acquisition time (s)	186 (47)	180 (50)	218 (45)	< 0.001***	0.102	< 0.001***	< 0.001***
Total examination time (s)	218 (48)	228 (56)	254 (53)	< 0.001***	< 0.001***	< 0.001***	< 0.001***

All values are presented as the median (interquartile range). A *p*-value of < 0.05 was considered statistically significant. *p*-value^a^ represents the comparison between BM-MRCP and RG-MRCP, *p*-value^b^ represents the comparison between RG-MRCP and NT-MRCP, and *p*-value^c^ represents the comparison between BM-MRCP and NT-MRCP

*MRCP* magnetic resonance cholangiopancreatography, *BM-MRCP* BioMatrix MRCP, *RG-MRCP* respiratory-gating MRCP, *NT-MRCP* navigator-triggered MRCP

*** *p* < 0.001

The total examination times for the three techniques were 218 (48) seconds for BM-MRCP, 228 (56) seconds for RG-MRCP and 259 (53) seconds for NT-MRCP was significantly shorter than both RG-MRCP and NT-MRCP.

### Qualitative analysis

**Fig. 3:**
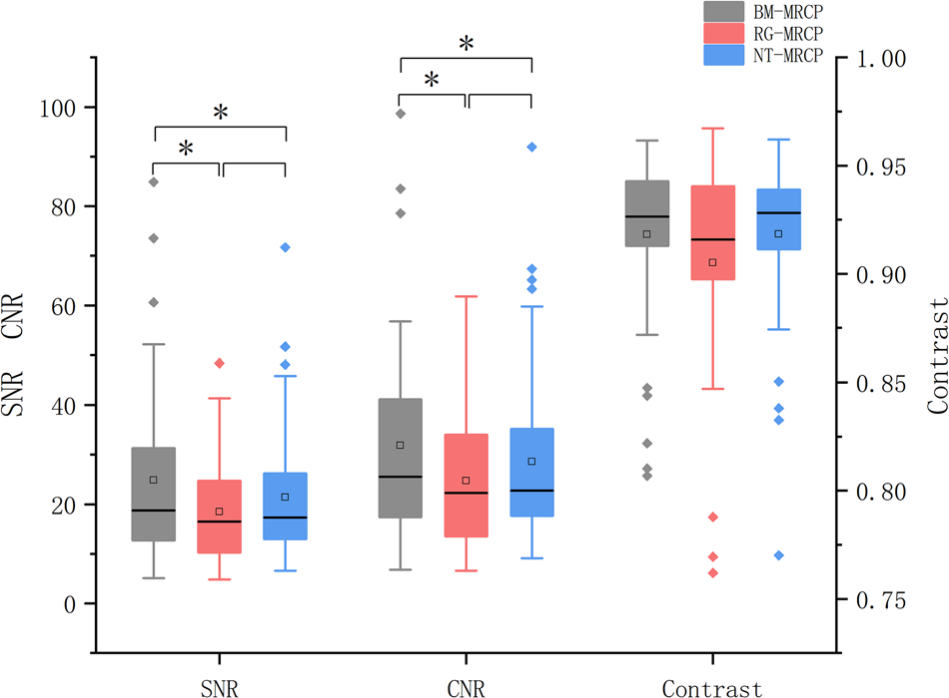
Comparisons of SNR, contrast, and CNR among three gating methods for 3D-MRCP. BM-MRCP showed significantly higher SNR and CNR than RG-MRCP and NT-MRCP, while contrast was comparable across groups. BM-MRCP also demonstrated a more favorable outlier distribution, suggesting finer image granularity. SNR, signal-to-noise ratio; CNR, contrast-to-noise ratio; MRCP, magnetic resonance cholangiopancreatography; BM-MRCP, BioMatrix MRCP; RG-MRCP, respiratory-gating MRCP; NT-MRCP, navigator-triggered MRCP

The qualitative analysis results are summarized in Table 2 and Fig. 3. BM-MRCP and NT-MRCP were significantly superior to RG-MRCP in visualizing the right and left peripheral hepatic ducts, right and left hepatic ducts, common hepatic duct, common bile duct, cystic duct, gallbladder, and main pancreatic duct. Additionally, BM-MRCP and NT-MRCP exhibited fewer artifacts and higher overall image quality compared to RG-MRCP. No significant differences between BM-MRCP and NT-MRCP in terms of duct visibility, artifact levels, or overall image quality. Interobserver consistency of image quality, assessed using the ICC, demonstrated strong consistency. Detailed results are presented in Table 3.

**Table 2:** Qualitative analysis of image quality for three MRCP methods (*n* = 45)

	BM-MRCP	RG-MRCP	NT-MRCP	*p*-value	*p*-value^a^	*p*-value^b^	*p*-value^c^
Left peripheral hepatic duct	3 (1)	2 (2)	3 (1)	< 0.001***	< 0.001***	< 0.001***	0.562
Right peripheral hepatic duct	3 (1)	2 (1.67)	3 (1)	< 0.001***	< 0.001***	< 0.001***	0.673
Left hepatic duct	3.67 (1)	3 (0.67)	3.67 (1)	< 0.001***	< 0.001***	< 0.001***	0.874
Right hepatic duct	3.33 (1)	3 (1)	3 (1)	< 0.001***	< 0.001***	0.001**	0.527
Common hepatic duct	4 (1)	3 (1)	4 (1)	0.006**	0.051	0.092	0.792
Common bile duct	4 (1)	3 (1)	4 (1)	< 0.001***	0.031*	0.035*	0.958
Cystic duct	3 (1)	3 (1)	3 (1)	< 0.001***	0.004**	0.020*	0.580
Gallbladder (*n* = 44)	4 (0)	3.33 (1)	4 (0.33)	< 0.001***	0.001**	0.003**	0.790
Main pancreatic	3 (1.33)	2.33 (1.67)	3 (1.33)	< 0.001***	0.001**	0.004**	0.527
Artifacts	3 (0)	3 (1)	3 (0.33)	< 0.001***	0.004**	0.005**	0.916
Overall rating	3 (1)	3 (1)	3 (1)	< 0.001***	< 0.001***	0.001**	0.833

All values are presented as median (interquartile range). A *p*-value of < 0.05 was considered statistically significant. Gallbladder (*n* = 44): One case of post-cholecystectomy is excluded. *p*-value^a^ indicates the comparison between BM-MRCP and RG-MRCP, *p*-value^b^ indicates the comparison between RG-MRCP and NT-MRCP, and *p*-value^c^ indicates the comparison between BM-MRCP and NT-MRCP

*MRCP* magnetic resonance cholangiopancreatography, *BM* BioMatrix, *RG* respiratory gating, *NT* navigator-triggered

* *p* < 0.05, ** *p* < 0.01, *** *p* < 0.001

**Table 3:** Consistency evaluation of qualitative analysis of image quality among three evaluators (*n* = 45)

	BM-MRCP		RG-MRCP		NT-MRCP	
	ICC (95% CI)	*p*-value	ICC (95% CI)	*p*-value	ICC (95% CI)	*p*-value
Left peripheral hepatic duct	0.908 (0.855–0.945)	< 0.001***	0.932 (0.890–0.960)	< 0.001***	0.867 (0.789–0.920)	< 0.001***
Right peripheral hepatic duct	0.953 (0.925–0.972)	< 0.001***	0.921 (0.876–0.953)	< 0.001***	0.915 (0.863–0.950)	< 0.001***
Left hepatic duct	0.962 (0.939–0.978)	< 0.001***	0.866 (0.792–0.919)	< 0.001***	0.926 (0.883–0.956)	< 0.001***
Right hepatic duct	0.943 (0.909–0.966)	< 0.001***	0.885 (0.815–0.932)	< 0.001***	0.940 (0.903–0.964)	< 0.001***
Common hepatic duct	0.932 (0.892–0.960)	< 0.001***	0.929 (0.886–0.958)	< 0.001***	0.866 (0.793–0.919)	< 0.001***
Common bile duct	0.928 (0.886–0.957)	< 0.001***	0.925 (0.881–0.955)	< 0.001***	0.965 (0.944–0.980)	< 0.001***
Cystic duct	0.960 (0.935–0.976)	< 0.001***	0.898 (0.834–0.940)	< 0.001***	0.952 (0.924–0.972)	< 0.001***
Gallbladder (*n* = 44)	0.966 (0.945–0.980)	< 0.001***	0.836 (0.737–0.903)	< 0.001***	0.905 (0.849–0.943)	< 0.001***
Main pancreatic duct	0.831 (0.742–0.897)	< 0.001***	0.886 (0.812–0.933)	< 0.001***	0.877 (0.808–0.926)	< 0.001***
Artifacts	0.978 (0.965–0.987)	< 0.001***	0.970 (0.952–0.983)	< 0.001***	0.980 (0.968–0.989)	< 0.001***
Overall rating	0.979 (0.966–0.988)	< 0.001***	0.978 (0.965–0.987)	< 0.001***	0.981 (0.969–0.989)	< 0.001***

*p* < 0.05 is considered statistically significant. Gallbladder (*n* = 44): One case of post-cholecystectomy is excluded

*ICC* intraclass correlation coefficient, *CI* confidence interval, *MRCP* magnetic resonance cholangiopancreatography, *BM* BioMatrix, *RG* respiratory-gating, *NT* navigator-triggered

*** *p* < 0.001

### Quantitative analysis

The quantitative analysis results are summarized in Table 4 and Fig. 3. BM-MRCP outperformed RG-MRCP and NT-MRCP in SNR and CNR. However, no significant differences in contrast were observed among the three techniques. The interobserver consistency for SNR, contrast, and CNR was 0.85 (95% CI: 0.78–0.91), 0.77 (95% CI: 0.68–0.87), and 0.90 (95% CI: 0.85–0.94), respectively, indicating good to excellent consistency. These results demonstrate the high reliability of the quantitative measurements.

**Table 4:** Comparison of SNR, contrast, and CNR among three gating methods for 3D-MRCP images

	BM-MRCP	RG-MRCP	NT-MRCP	*p*-value	*p*-value^a^	*p*-value^b^	*p*-value^c^
SNR	18.76 (18.64)	16.48 (14.45)	17.34 (13.29)	< 0.001***	< 0.001***	0.114	< 0.001***
Contrast	0.93 (0.03)	0.92 (0.04)	0.93 (0.03)	0.083	N/A	N/A	N/A
CNR	25.54 (23.79)	22.24 (20.50)	22.74 (17.54)	< 0.001***	< 0.001***	0.171	0.003**

All values are presented as median (interquartile range). A *p*-value of < 0.05 was considered statistically significant. *p*-value^a^ indicates the comparison between BM-MRCP and RG-MRCP, *p*-value^b^ indicates the comparison between RG-MRCP and NT-MRCP, and *p*-value^c^ indicates the comparison between BM-MRCP and NT-MRCP

*SNR* signal-to-noise ratio, *CNR* contrast-to-noise ratio, *MRCP* magnetic resonance cholangiopancreatography, *BM-MRCP* BioMatrix MRCP, *RG-MRCP* respiratory-gating triggered using respiratory bellows, *NT-MRCP* navigator-triggered MRCP, *N/A* not applicable

** *p* < 0.01, *** *p* < 0.001

### Technical failures

Two patients (4.3%) failed RG-MRCP or NT-MRCP due to shallow or deep breathing (failure rates of 2.1% for both RG-MRCP and NT-MRCP), while BM-MRCP had no technical failures. These findings warrant further validation in larger patient cohorts.

## Discussion

This study compared and evaluated the performance of three free-breathing 3D-MRCP techniques: BM, RG, and NT. The findings demonstrated that BM-MRCP offers significant advantages in scanning efficiency, image quality, and artifact control. The image acquisition time for BM-MRCP was significantly shorter than that of NT-MRCP, and the total examination time was markedly reduced compared to both RG-MRCP and NT-MRCP. Additionally, BM-MRCP demonstrated greater stability in visualizing biliary system structures and yielded higher signal-to-noise ratio (SNR) and contrast-to-noise ratio (CNR). These results provide valuable insights for optimizing clinical biliary imaging techniques.

During relaxed breathing, the human diaphragm typically moves vertically by 1–3 cm [18–20]. Effective respiratory motion management is essential for high-quality MRCP imaging. Studies have shown that combining various sequences with breath-holding can significantly improve scan speed without compromising image quality [10–13, 21, 22]. However, patients with hearing impairments, reduced pulmonary function, advanced age, or severe illness, as well as pediatric patients, often struggle to hold their breath. To address this limitation, we conducted 3D-MRCP scans under free-breathing conditions using three different gating methods (BM, RG, and NT) in the same patient cohort to compare imaging efficiency and quality. To our knowledge, no prior studies have conducted such a systematic evaluation.

The NT methods detect stable respiratory waveforms to establish the breathing cycle, necessitating additional RF and gradient pulses and real-time motion signal processing, which significantly prolongs scan times [23–25]. The irregularity and dynamic changes in diaphragm position further complicate triggering and data acquisition, limiting NT’s scanning efficiency [26]. NT also relies heavily on motion compensation algorithms, and the real-time processing demands exacerbate the computational burden, further extending scan times [27]. Our findings showed that BM-MRCP and RG-MRCP required less acquisition time than NT-MRCP, with BM-MRCP reducing the average acquisition time by 11.95% compared to NT-MRCP, consistent with theoretical expectations.

RG-MRCP uses an external breathing belt to detect abdominal motion, converting physical deformation into a voltage waveform for MRI gating. However, belt placement requires patient cooperation, which can be time-consuming [15]. NT-MRCP, while not reliant on external devices, necessitates the placement of diaphragm navigation bars in multiple orientations and additional navigation training before scanning, increasing the total examination time. In addition, cardiac motion may also interfere with navigator stability, particularly in cases where the diaphragm tracking position is anatomically close to the heart. By contrast, BM-MRCP detects respiratory motion through impedance changes in RF coils integrated into the spine coil, eliminating the need for external devices [28, 29]. Once the patient is positioned, BM reconstructs the breathing waveform and initiates the scan immediately, significantly reducing total examination time. BM-MRCP reduced the average total examination time by 6.30% and 13.81% compared to RG-MRCP and NT-MRCP, respectively. This was attributed to BM’s optimized workflows and enhanced acquisition efficiency.

**Fig. 4:**
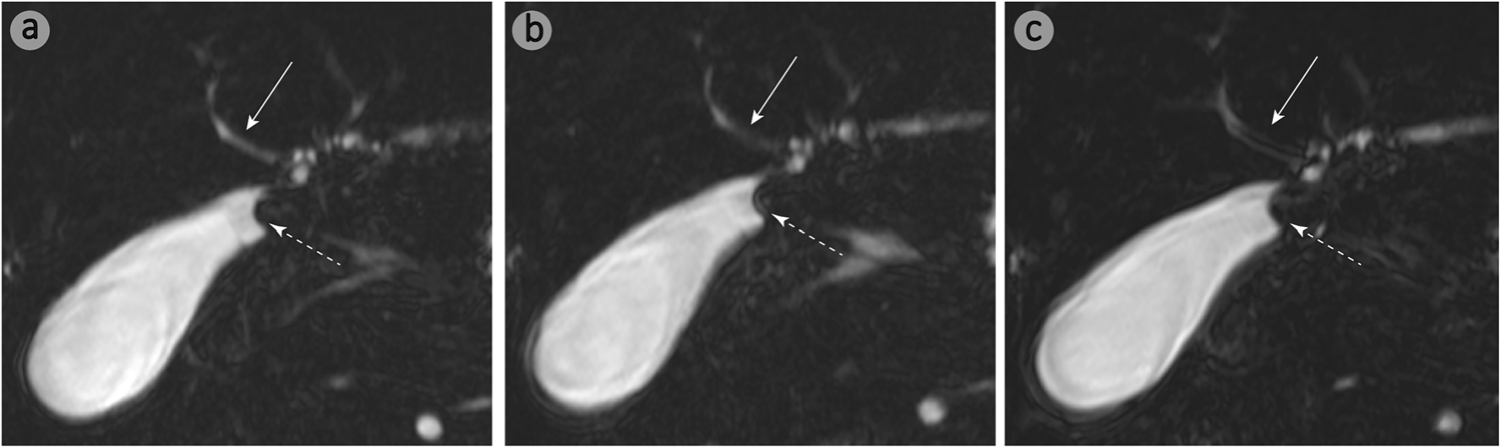
A 31-year-old female with surgically confirmed gallstones near the gallbladder neck. BM-MRCP (**a**) provides a clearer visualization of the gallstone margin (dashed arrow) and the right hepatic duct (solid arrow) compared to RG-MRCP (**b**) and NT-MRCP (**c**). RG-MRCP shows blurred gallstone margins, while NT-MRCP exhibits bilateral artifacts in the right hepatic duct, impairing diagnostic assessment. BM-MRCP, BioMatrix MRCP; RG-MRCP, respiratory-gating MRCP using respiratory bellows; NT-MRCP, navigator-triggered MRCP

**Fig. 5:**
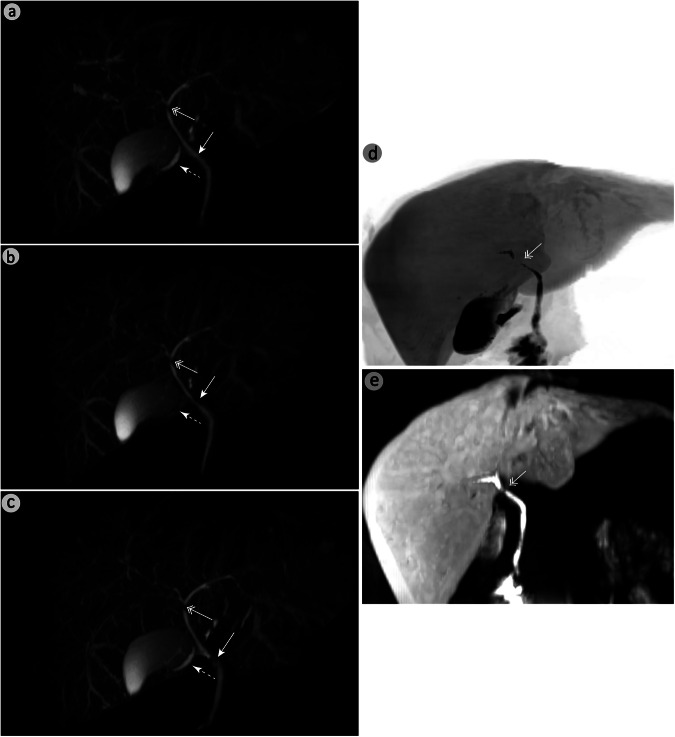
A 26-year-old male with suspected cholangitis. BM-MRCP (**a**) clearly shows common hepatic duct narrowing (double-headed arrow) and delineates both the cystic (dashed arrow) and common bile ducts (solid arrow). RG-MRCP (**b**) shows blurred cystic duct margins and continuous but indistinct narrowing. NT-MRCP (**c**) shows discontinuity in the common hepatic duct and obscured common bile duct due to artifacts. Hepatocyte-specific contrast-enhanced MRCP (**d**, **e**) confirmed the narrowing and patency findings. BM-MRCP, BioMatrix MRCP; RG-MRCP, respiratory-gating MRCP using respiratory bellows; NT-MRCP, navigator-triggered MRCP

RG methods rely on external belts for respiratory signals, but factors such as patient body size, incorrect belt placement, or poor compliance can produce ineffective signals that fail to reflect the respiratory plateau phase, leading to inaccurate respiratory motion estimation [30–32]. This reduces image quality [33, 34]. BM-MRCP, which does not require external devices, avoids such issues. Furthermore, RG methods trigger data acquisition only after receiving a signal, limiting continuous acquisition and reducing image SNR and CNR [26]. Consequently, RG-MRCP frequently exhibits artifacts under high respiratory amplitude or rapidly fluctuating respiratory patterns [14], as shown in Figs. 4 and 5. In contrast, BM-MRCP dynamically captures and corrects respiratory motion in real-time, producing clearer images even under complex conditions. Our study demonstrated that BM-MRCP was superior to RG-MRCP in visualizing the biliary system, suppressing artifacts, and achieving overall image quality, except for the CBD. Quantitative analysis of SNR and CNR confirmed that BM-MRCP’s superiority in artifact control and imaging consistency.

NT relies on real-time acquisition of diaphragm position signals for triggering. However, processing delays during signal acquisition can result in loss of critical dynamic signals, potentially compromising temporal resolution, signal continuity, and dynamic range, leading to reduced SNR [35, 36]. NT is also prone to significant motion artifacts under irregular breathing or mismatched motion conditions, particularly in high-field MRI environments [35, 37]. Additionally, NT requires supplementary RF and gradient pulses, and navigation echoes sharing frequencies with MRI signals may interfere with imaging, reducing SNR and increasing artifacts [35, 36, 38]. Although qualitative analysis found no significant difference in image quality between BM-MRCP and NT-MRCP, quantitative analysis of SNR and CNR demonstrated that BM-MRCP was significantly superior. In some cases, NT-MRCP artifacts lowered diagnostic confidence (Figs. 4, 5), while BM-MRCP provided clearer images with fewer artifacts, enhancing diagnostic value.

**Fig. 6:**
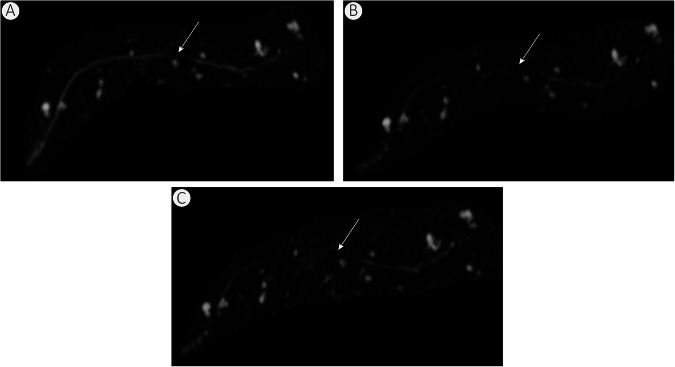
Female patient, 54 years old, with incidentally detected intraductal papillary mucinous neoplasm (IPMN). Maximum intensity projection images from BM-MRCP (**A**), RG-MRCP (**B**), and NT-MRCP (**C**) all demonstrate the lesion; however, BM-MRCP shows clearer delineation of the main pancreatic duct compared to the other two techniques

Our prospective study presents representative cases (Figs. 4–6) where BM-MRCP showed fewer artifacts and clearer delineation of biliary and pancreatic ducts than RG- and NT-MRCP. These findings provide preliminary evidence that its superior image quality may improve diagnostic confidence, though large-scale, disease-specific studies are needed to verify improvements in diagnostic accuracy and clinical outcomes.

BioMatrix technology is an innovative motion-sensing approach using self-resonant RF coils embedded in the spine array (Supplementary Fig. 2). These coils independently detect respiratory motion, minimizing interference and improving efficiency [17]. By wirelessly capturing motion, BM reduces delays and asynchrony associated with conventional gating, enhancing abdominal image SNR and CNR [39]. Prior studies have shown higher liver and abdominal SNR with BM than NT [28]. In our study, we also observed improvements in motion management with BM, which led to better MRCP image quality under complex motion conditions. Moreover, BM’s real-time motion detection offers superior speed and precision compared to RG methods [37], and its high spatiotemporal resolution helps preserve tissue details in challenging motion environments, making it particularly suitable for high-field imaging and fine motion correction [35].

Two patients failed (4.3%) to complete RG- or NT-MRCP due to shallow or deep-slow breathing, resulting in a failure rate of approximately 2.13% for RG-MRCP and NT-MRCP. In contrast, BM-MRCP successfully completed scans for all 47 patients, demonstrating its ability to operate stably under a wider range of breathing conditions and highlighting its greater versatility and robustness. However, due to the limited sample size, this result should be interpreted cautiously and confirmed by larger studies.

Different gating methods may benefit distinct patient groups based on breathing patterns, liver function, and compliance. While this study focused on free-breathing methods, breath-hold (MRCP-BH) using compressed sensing has shown excellent image quality in selected patients with adequate cooperation. Future comparative studies involving MRCP-BH and BM-MRCP, particularly in populations with poor compliance or impaired respiratory control, would help refine individualized MR imaging strategies in clinical practice.

Potential confounders, such as patient age, BMI, and underlying respiratory or neurological conditions, could influence breathing patterns and therefore impact the imaging quality or scan time. In this study, we minimized the effects of inter-individual variability by using an intra-subject design, in which each patient underwent all three MRCP techniques in randomized order. However, no formal statistical adjustment was made for these confounders. Future studies with larger cohorts may consider stratifying the patient population by these factors to further refine the findings and adjust for potential biases.

The clinical significance of BM-MRCP is particularly evident in its ability to meet the needs of patient populations with poor compliance, such as those with hearing impairments, critically ill patients, individuals with impaired pulmonary function, and pediatric patients. As a non-contact, respiratory-triggered technology, BM-MRCP eliminates the need for additional physical contact with the patient, such as the use of a respiratory belt, which is especially important for these patient groups. This feature reduces discomfort and enhances patient comfort. Moreover, the simplicity of BM-MRCP significantly reduces patient preparation time and streamlines the imaging acquisition process (e.g., eliminating the need for navigator placement and pre-learning processes), making it particularly well-suited for high-throughput clinical environments. In terms of image quality, BM-MRCP can capture and correct respiratory artifacts in real-time, improving the visualization of the pancreatic and biliary ducts, as well as other important fluid-filled structures, ensuring higher diagnostic accuracy.

Emerging studies suggest that the new version of BioMatrix (Pilot Tone Navigator) can simultaneously differentiate cardiac and respiratory motions, enabling free-breathing, cardiac-respiratory-resolved five-dimensional (5D) MRI reconstruction [28, 36]. This sequence-independent approach eliminates the need for navigational readouts or periodic SI projections [40], optimizing workflows and enhancing k-space trajectory design flexibility. Although BM is relatively new to clinical practice, our results support its broader adoption.

Overall, BM-MRCP not only enhances clinical workflow efficiency but also significantly improves patient experience, making it of great clinical value, especially in situations requiring rapid and accurate imaging.

### Limitations

This study has several limitations. First, it employed proprietary respiratory-gating technology from a single MRI vendor, which may limit the generalizability of our findings to other platforms or systems. Second, the relatively small sample size and single-center design may introduce statistical bias, and larger, multicenter studies are warranted for further validation. Third, although our primary analysis focused on image quality and workflow efficiency, we did not perform subgroup comparisons across different pancreaticobiliary pathologies (e.g., stones, strictures, or neoplasms) due to limited and uneven disease distribution. As such, the diagnostic performance of each technique in specific disease categories remains unassessed and should be systematically investigated in future studies with stratified designs and adequate statistical power.

## Conclusion

BM-MRCP acquires high-quality images in a shorter time under free-breathing conditions, demonstrating significant advantages over RG-MRCP and NT-MRCP. Its non-contact nature, high precision, and operational simplicity provide a solid foundation for widespread clinical adoption and contribute to the advancement of MRI technology.

## Supplementary information

ELECTRONIC SUPPLEMENTARY MATERIAL

## Data Availability

The data that support the findings of this study are available from Y.Q. Restrictions apply to the availability of these data, which were used under license for the current study and therefore are not publicly available. Data are, however, available from the authors upon reasonable request and with permission from Y.Q.

## Abbreviations

3D-MRCP: Three-dimensional magnetic resonance cholangiopancreatography
BM: BioMatrix
CBD: Common bile duct
CNR: Contrast-to-noise ratio
ICC: Intraclass correlation coefficient
NT: Navigator-triggered
RF: Radiofrequency
RG: Respiratory gating using respiratory bellows
ROI: Region of interest
SD: Standard deviation
SI: Signal intensity
SNR: Signal-to-noise ratio
