# Supplementary material for: Advantages of BioMatrix respiratory gating in free-breathing three-dimensional magnetic resonance cholangiopancreatography: a prospective comparative study
Source: Insights Imaging. 2025 Jun 27;16:137. doi: 10.1186/s13244-025-02023-4 (PMC12205134; doi:10.1186/s13244-025-02023-4)
Supplement: Supplementary file 1 — ELECTRONIC SUPPLEMENTARY MATERIAL [file 13244_2025_2023_MOESM1_ESM.pdf]

Advantages of Biomatrix Respiratory Gating in Free-Breathing Three-dimensional Magnetic Resonance Cholangiopancreatography: A Prospective Comparative Study

ELECTRONIC SUPPLEMENTARY MATERIAL

Suppl_Figures. 1– Representative images demonstrating ROI placement for quantitative measurements. Signal intensity (SI) was measured in the common bile duct (red circle), periductal tissues (yellow circle), and liver parenchyma (green circle). The standard deviation (SD) of signal intensity within the same ROIs was used to estimate image noise.

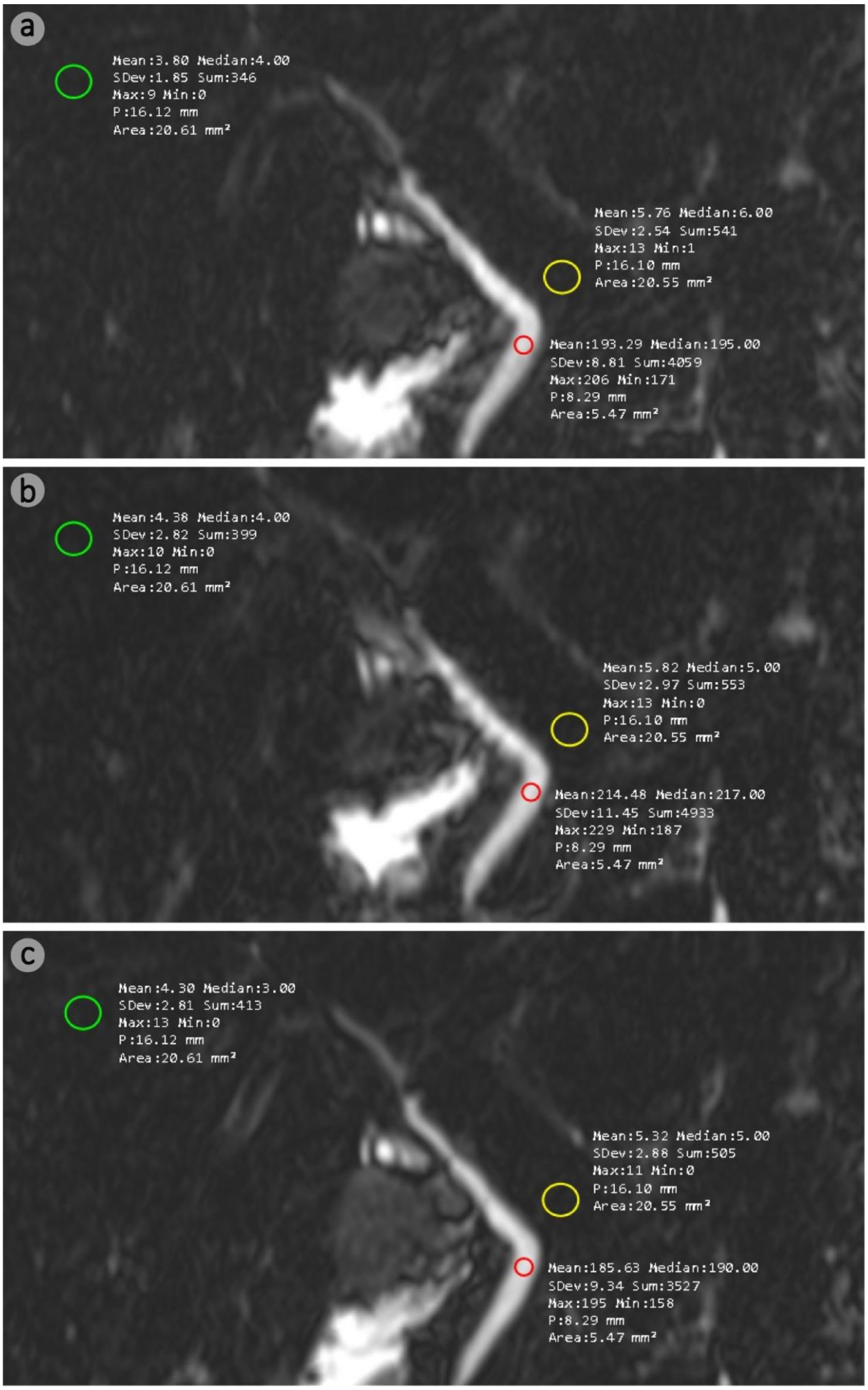

(a) BM-MRCP; (b) RG-MRCP; (c) NT-MRCP.

SI = Signal Intensity; SD = Standard Deviation; ROI = Region of Interest; CBD = Common Bile Duct; BM-MRCP = Biomatrix MRCP; RG-MRCP = Respiratory-gating MRCP; NT-MRCP = Navigator-triggered MRCP.

Insights Imaging (2025) Qing Y, Xueyi D, Qiuyang G, et al.

Suppl_Figures. 2– Schematic illustration of the respiratory signal generation mechanism in the first-generation BioMatrix system (configured on 3.0T MAGNETOM Vida, Siemens). Self-resonant RF coils (ωBM = 30 MHz), integrated into the spine coil array, generate a near-field magnetic field (BBM) modulated by respiratory motion-induced changes in tissue distribution. Respiratory and MRI signals are independently acquired by the self-resonant coils and conventional multi-channel receive coils, respectively, and used for scan control and image reconstruction. Red lines represent MR receive coils and data links; yellow lines indicate BioMatrix self-resonant coils and transmission links; green frames depict integrated data processing for respiratory wave generation and image reconstruction.

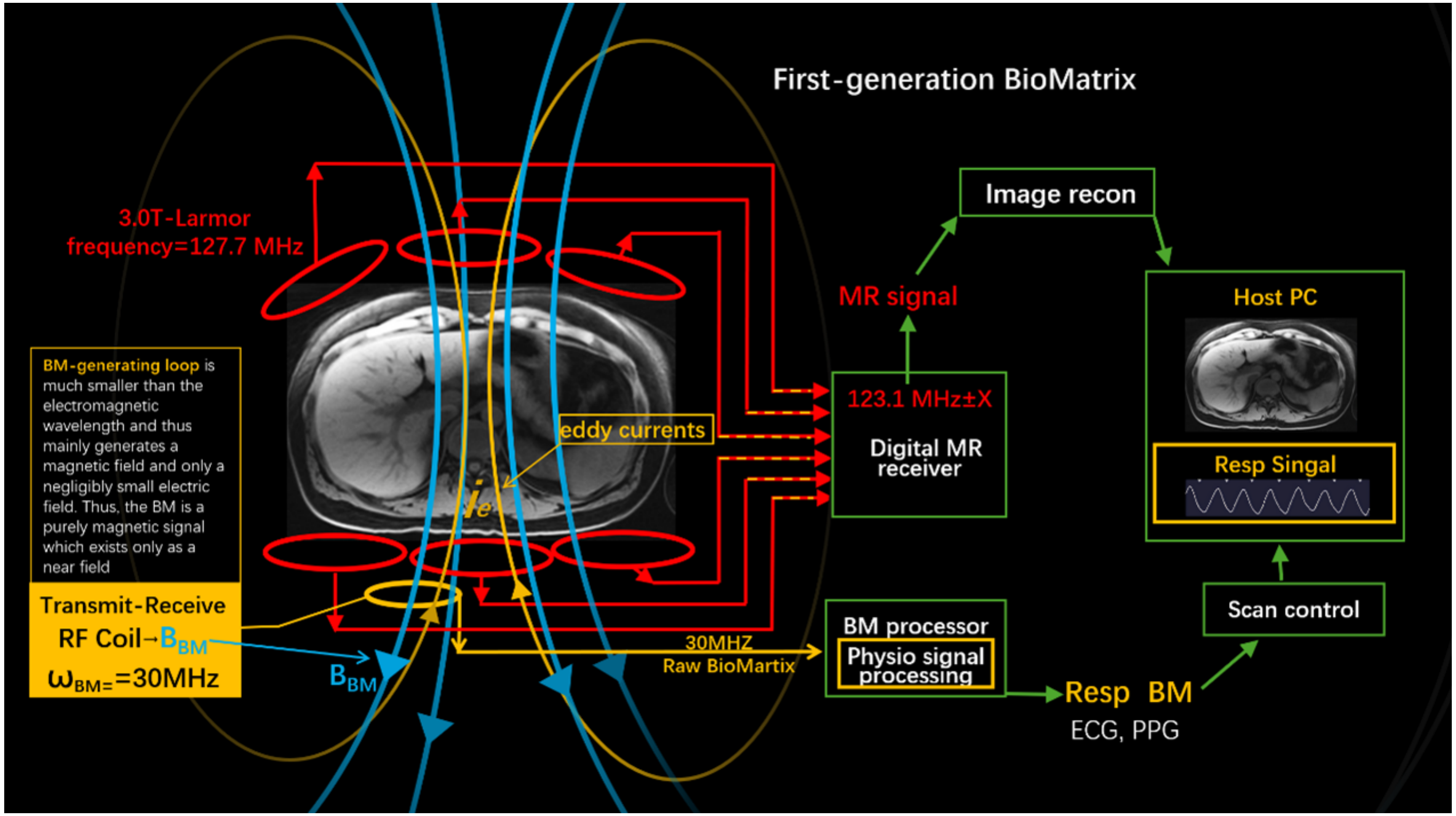

RF = radio frequency; ECG = electrocardiogram; PPG = photoplethysmograph.
